# Carcinoma En Cuirasse: A Rare but Striking Cutaneous Manifestation of Metastatic Breast Cancer

**DOI:** 10.7759/cureus.39838

**Published:** 2023-06-01

**Authors:** Manasi Godbole, Kashmira Wani, Shereen Zia, Vrushali Dabak

**Affiliations:** 1 Hematology/Medical Oncology, Henry Ford Health System, Detroit, USA; 2 Internal Medicine, Henry Ford Health System, Detroit, USA; 3 Pathology, Henry Ford Health System, Detroit, USA

**Keywords:** invasive ductal carcinoma, skin lesions, estrogen-receptor positive, her2-positive, metastatic breast cancer

## Abstract

Carcinoma en cuirasse is a rare cutaneous metastatic presentation of breast cancer with a poor prognosis. We report a female in her 70s with a prior history of left breast ductal carcinoma in situ status post-radiation and lumpectomy who presented with skin thickening of the left breast and a few solid masses in bilateral breasts. Biopsy showed invasive ductal carcinoma of the left breast (estrogen receptor [ER]/progesterone receptor positive [PR], human epidermal growth factor receptor-2 [HER2] negative) and ductal carcinoma in situ of the right breast (ER/PR positive). She underwent a right breast lumpectomy; however, the left breast mastectomy was aborted due to the worsening of her skin findings on preoperative examination. A skin biopsy revealed poorly differentiated invasive ductal carcinoma. She was diagnosed with stage 4 breast cancer, specifically carcinoma en cuirasse. Systemic treatment was initiated, followed by a left breast mastectomy. A surgical biopsy was HER2-positive, and therefore anti-HER2 therapy was given. She remains on maintenance therapy with an excellent response at present.Any unexplained skin findings in breast cancer patients should prompt consideration of carcinoma en cuirasse. With ongoing treatment advances, many newer therapy options are available for metastatic breast cancer. Based on our case, we think that patients with this disease can have better outcomes.

## Introduction

Carcinoma en cuirasse is a rare cutaneous presentation most noted to have metastasized from primary breast cancers; however, cases have also been reported from the gastrointestinal tract [1] or lung [2] sites. Among patients with breast cancer, this phenomenon is typically seen months to years after a mastectomy [3]. A retrospective study demonstrated it to be the first sign of cancer in 59 of 7316 breast cancer patients, highlighting that unexplained skin involvement should not be ignored [4].

## Case presentation

A 70-year-old white female with a past medical history of diabetes mellitus, hypertension, osteoarthritis, psoriasis, left breast ductal carcinoma in situ status post-radiation, and lumpectomy in 2007 presented 10 years later with irregularities in both her breasts noted on a diagnostic mammogram. Previous mammograms post-lumpectomy had shown fibro-glandular densities but no masses, calcifications, or architectural distortion. A subsequent breast ultrasound showed a 7 mm × 4 mm × 8 mm solid mass in her right breast at the 8 o'clock position and a 12 mm × 11 mm × 12 mm solid mass in her left breast with irregular margins at the 2 to 3 o'clock position. Of note, skin thickening was noted on the left breast examination; however, an ultrasound of that area did not reveal solid or cystic masses. No lymphadenopathy was noted on axillary ultrasound. Also, the patient had a long-standing history of psoriasis, to which these skin findings were initially attributed. Furthermore, she had a history of left breast cellulitis in 2015, for which she was admitted to the hospital, treated with intravenous antibiotics, and discharged on a 10-day course of doxycycline.

**Figure 1:**
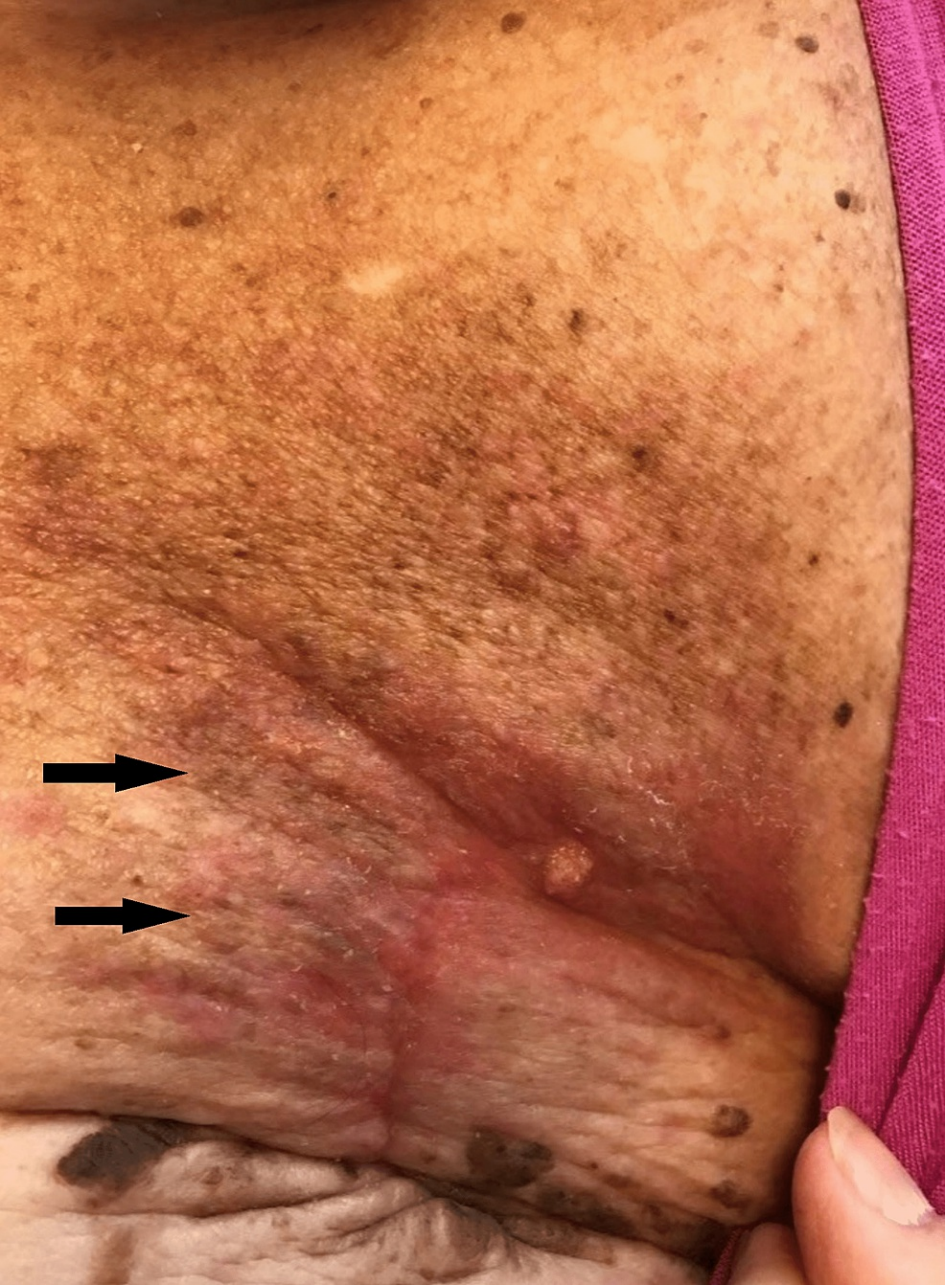
Skin findings on presentation. Consent obtained from the patient prior to submitting in the manuscript.

Ultrasound-guided needle biopsy of the masses confirmed moderately differentiated invasive ductal carcinoma of the left breast (estrogen receptor [ER]/progesterone receptor [PR] positive, human epidermal growth factor receptor-2 [HER2] equivocal by immunohistochemistry testing [negative by fluorescence in situ hybridization test]), as well as an intraductal papilloma of the right breast, intermediate grade ductal carcinoma in situ (ER/PR positive). She had a successful right breast lumpectomy two months later, with plans to also undergo a left breast mastectomy for recurrent disease in that same procedure. Unfortunately, the planned mastectomy was not completed due to the worsening of her skin thickening, with now over a dozen papules noted on her left breast during the preoperative examination (Figure 1).

**Figure 2:**
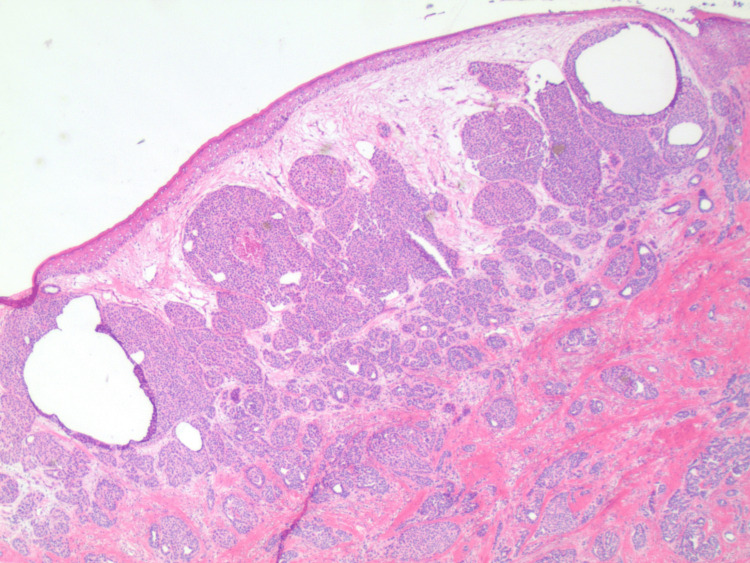
Poorly differentiated invasive ductal carcinoma involving superficial and deep dermis and focally involving the epidermis (2× magnification, ER stain). ER stain: estrogen receptor stain seen as deep blue color throughout the slide image highlighting positive uptake in epidermis, superficial and deep dermis.

An excisional biopsy of the skin was taken, which later revealed poorly differentiated invasive ductal carcinoma involving the dermis, deep dermis, and focally involving the epidermis (Figure 2).

**Figure 3:**
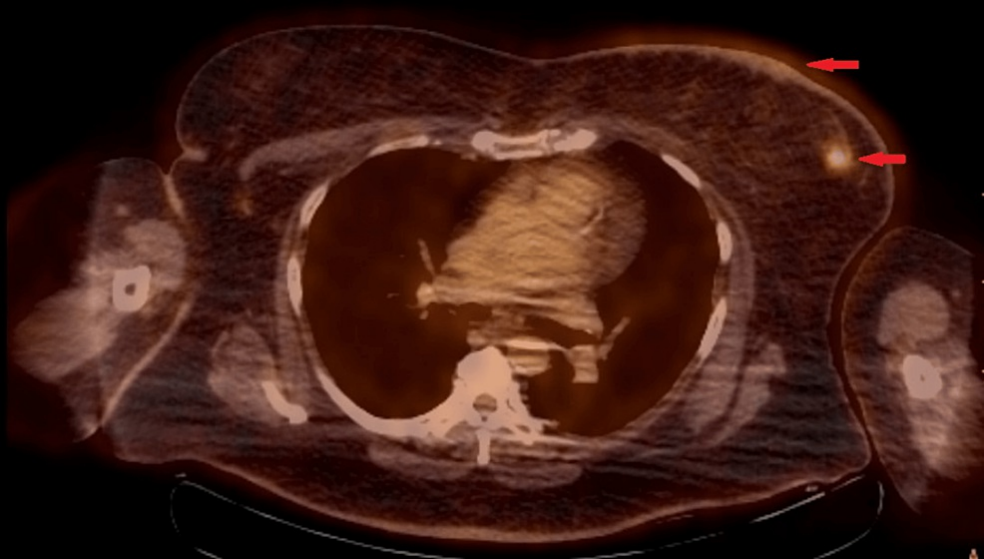
PET-CT image showing fluorodeoxyglucose avid left breast skin and parenchymal lesions (red arrows). Consent obtained from patient prior to submitting in the manuscript. Mildly distorted image due to motion artifact.

Due to metastasis to the skin, what was previously thought to be stage 1 breast cancer is now stage 4 breast cancer, and more specifically, carcinoma en cuirasse. A positron emission tomography-computed tomography scan was consistent with these findings, showing biopsy-proven left breast malignancy as well as metabolic activity within the left breast parenchyma and skin (Figure 3). BRCAplus genetic testing was positive for the CHEK2 gene (p.I157T) and otherwise negative for other mutations (BRCA 1/2, ATM, CDH1, PALB2, PTEN, and TP53).

**Figure 4:**
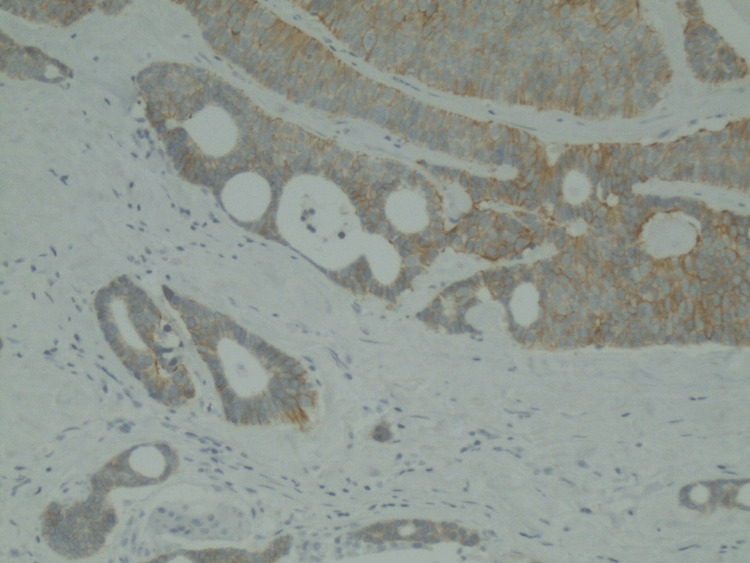
HER2 receptor staining (10× magnification) on left breast mastectomy surgical specimen.

Her treatment was initiated with palbociclib (125 mg) and letrozole (2.5 mg) for metastatic hormone receptor-positive breast cancer. She developed neutropenia after cycle 1, but then completed seven total cycles of dose reduction. Given the dramatic clinical response and radiological response noted on subsequent scans, she later underwent a left breast mastectomy. The surgical specimen showed residual disease in the lymph nodes as well as dermal and angiolymphatic invasion. Thus, she continued to receive hormonal treatment with palbociclib and letrozole for two more cycles. Interestingly, the HER2 receptor was reported as positive on the surgical specimen of the left breast (Figure 4).

Her treatment, therefore, was switched to a regimen consisting of docetaxel, trastuzumab, and pertuzumab. Docetaxel was discontinued after a few cycles due to adverse effects (diarrhea and leg swelling), and she was continued on trastuzumab and pertuzumab with the addition of letrozole for the next 55 cycles until 2021. Thereafter, pertuzumab was discontinued due to side effects (e.g., diarrhea), and only letrozole has been continued with trastuzumab at present. She is currently on cycle 78 and doing well five years later with negative positron emission tomography scans.

## Discussion

Under the microscope, carcinoma en cuirasse is described as a small number of tumor cells interspersed among bundles of collagen [5]. Macroscopically, areas of swelling and erythema of the skin, like cellulitis, are seen, similar to our patient’s multiple previous presentations/admissions. These areas later develop into thickened, rough patches of skin [5] and even nodules, like in our patient. Carcinoma en cuirasse usually manifests as skin lesions mainly over the chest wall or nearby abdominal wall, and sometimes even on the axillary folds and arms. Mahore et al. reported a case in which a 50-year-old female presented with nodular skin lesions, but she also had indurated skin like a keloid [3]. Salemnis et al. presented a case in which a 60-year-old woman developed a painful erythematous lesion that later became indurated [6]. The pathophysiology behind this phenomenon is thought to be attributed to the gene pleiotrophin, which is a protooncogene that can serve as the nidus for tumor formation [7]. Carcinoma en cuirasse holds a poor prognosis, and treatment of the disease is difficult [8]. Chemotherapeutic agents are unable to reach the tumor cells, and radiation is ineffective because of the curvature of the skin folds [5]. Kumar et al. have shown promising results from rotational subtotal skin electron beam therapy, in which 4400 rad were given in 22 fractions [5]. There was a substantial improvement both within the lesions themselves as well as the associated pruritis. This treatment, though, works best post-mastectomy, once any irregularities in the skin are removed. Siddiqui and Zaman showed that even 12 weeks of tamoxifen therapy can completely resolve lesions [9]. In another study, Cabula et al. showed a 58% complete response rate when chemotherapy (bleomycin specifically) was combined with electrical pulse activity [8]. Case reports have also shown the role of hormonal therapy in patients with ER/PR-positive tumors, like our patient who received letrozole [10]. Our case is unique as our patient did not have a mastectomy prior to the diagnosis of carcinoma en cuirasse. The patient was initially treated with letrozole and palbociclib as per guidelines for hormone-positive metastatic breast cancer with an excellent clinical response and later underwent a mastectomy that was therefore considered a part of her therapy. Another unique point about this case is the change in receptor status to become HER2-positive, which only happens 10-15% of the time [11]. The change in receptor status allowed her treatment regimen to include anti-HER2 therapy. We think that this has helped the patient stay on minimally toxic therapy, with prolonged survival of more than five years and ongoing therapy to date.

## Conclusions

Carcinoma en cuirasse is a rare entity; however, any unexplained skin findings, especially in patients with a history of and/or active breast cancer, should prompt consideration of this as a possible differential diagnosis for appropriate staging and treatment considerations. With many new therapy options available to treat metastatic breast cancer according to hormone receptor status as well as HER2 receptor status, this disease can be treated effectively with excellent long-term outcomes, as highlighted in our case report.
